# TRPV1 Antagonists as Novel Anti-Diabetic Agents: Regulation of Oral Glucose Tolerance and Insulin Secretion Through Reduction of Low-Grade Inflammation?

**DOI:** 10.3390/medsci7080082

**Published:** 2019-07-24

**Authors:** Dorte X. Gram, Josefine Fribo, Istvan Nagy, Carsten Gotfredsen, Ana Charrua, John B. Hansen, Anker J. Hansen, Arpad Szallasi

**Affiliations:** 1 PILA Pharma, 211 21 Malmö, Sweden; 2 Former Employee, Research & Development, Novo Nordisk A/S, 2760 Måløv, Denmark; 3 Department of Anaesthetics, Pain Medicine and Intensive Care, Imperial College, London SW7 2AZ, UK; 4 Institute of Histology and Embryology, Faculty of Medicine, University of Porto, Alameda Prof Hernani Monteiro, 4099-002 Porto, Portugal; 5 Concit Pharma APS, 4450 Jyderup, Denmark; 6 1st Department of Pathology and Experimental Cancer Research, Semmelweis University, 1085 Budapest, Hungary

**Keywords:** vanilloid (capsaicin) receptor TRPV1, type-2 diabetes (T2DM), neurogenic inflammation, TRPV1 antagonist BCTC, glucose tolerance

## Abstract

With a global prevalence among adults over 18 years of age approaching 9%, Type 2 diabetes mellitus (T2DM) has reached pandemic proportions and represents a major unmet medical need. To date, no disease modifying treatment is available for T2DM patients. Accumulating evidence suggest that the sensory nervous system is involved in the progression of T2DM by maintaining low-grade inflammation *via* the vanilloid (capsaicin) receptor, Transient Receptor Potential Vanilloid-1 (TRPV1). In this study, we tested the hypothesis that TRPV1 is directly involved in glucose homeostasis in rodents. TRPV1 receptor knockout mice (*Trpv1*^−/−^) and their wild-type littermates were kept on high-fat diet for 15 weeks. Moreover, Zucker obese rats were given the small molecule TRPV1 antagonist, N-(4-Tertiarybutylphenyl)-4-(3-cholorphyridin-2-yl)tetrahydropyrazine-1(2H)-carbox-amide (BCTC), *per os* twice-a-day or vehicle for eight days. Oral glucose tolerance and glucose-stimulated insulin secretion was improved by both genetic inactivation (*Trpv1*^−/−^ mice) and pharmacological blockade (BCTC) of TRPV1. In the obese rat, the improved glucose tolerance was accompanied by a reduction in inflammatory markers in the mesenteric fat, suggesting that blockade of low-grade inflammation contributes to the positive effect of TRPV1 antagonism on glucose metabolism. We propose that TRPV1 could be a promising therapeutic target in T2DM by improving glucose intolerance and correcting dysfunctional insulin secretion.

## 1. Introduction

Type-2 diabetes (T2DM) is a chronic metabolic disorder that occurs when the β cells of the Langerhans islets cannot produce enough insulin to keep blood glucose within the physiological range, or when the body cannot effectively utilize the insulin that it produces [1]. The prevalence of T2DM is on the rise, especially in middle to low income countries, with a severe economic burden [2]. In 2014, the number of T2DM patients exceeded 420 million, killing 1.6 million every year [3]. Indeed, T2DM is a leading cause of blindness, renal failure, cardiovascular disease (CVD) including stroke, and limb amputation. The World Health Organization projects that T2DM will become the seventh leading cause of death worldwide by 2030. The cost of caring for T2DM patients is crippling: in 2012, Americans had spent $245 billion on the treatment of T2DM and its complications. This represents ~10% of the US health care spending.

An effective pharmacological treatment of T2DM should simultaneously normalize blood glucose levels, reduce body weight, and minimize the risk of developing CVD [1]. Unfortunately, the currently available pharmacological treatment options do not achieve all these goals. For example, there is no evidence that metformin, a first line T2DM drug, can reduce the risk for CVD [4]. Sodium-Glucose Co-transporter-2 (SGLT2) inhibitors (glifozins) were shown to reduce blood pressure in most, but not all, clinical studies [5]. Furthermore, treatment with insulin, sulfonylureas, and glinides has been associated with weight gain [6], whereas thiazolidinediones may increase the risk of congestive heart failure [7]. Since 2017, the SGLT2 inhibitor, empagliflozin (Jardiance), and the Glucagon-like Peptide-1 (GLP-1) agonist, liraglutide (Victoza), have been recommended as the second line treatment to patients with T2DM and established CVD who do not receive adequate regulation of their diabetes with metformin [8]. Common side-effects of empagliflozin include weakness, joint pain, and elevated cholesterol [9], whereas liraglutide may cause weakness and a variety of gastrointestional side-effects such as nausea and diarrhea. Clearly, there is an unmet need for novel antidiabetic drugs.

There is good experimental evidence that T2DM (along with obesity) has a low-grade inflammatory component [10], maintained at least in part by TRPV1-expressing sensory neurons [11]. Chemical ablation of these neurons by neonatal capsaicin administration (50 mg/kg subcutaneously, s.c.) improves insulin sensitivity in the rat [12]. Furthermore, desensitization of capsaicin-sensitive neurons prevents the development of spontaneous hyperglycemia in adult Zucker diabetic fatty (ZDF) rats [13]. Among neurotransmitters produced by capsaicin-sensitive neurons, calcitonin gene-related peptide (CGRP) seems to play a major role in T2DM (reviewed in [13]). Indeed, an increase in plasma CGRP precedes the development of obesity in Zucker obese rats [14]. CGRP, in turn, may inhibit insulin release from β cells [15] and initiate a harmful neurogenic inflammatory response in the pancreas [16].

As discussed above, the role of capsaicin-sensitive (TRPV1-expressing) sensory neurons in maintaining the low-grade chronic inflammation that underlies T2DM is well-established (reviewed in [13]). Less clear is the direct role of TRPV1 in the pathomechanism of T2DM. One has to keep in mind that capsaicin desensitization is not restricted to TRPV1. Indeed, capsaicin desensitization affects (“silences”) the whole sensory neuron with all the receptors that it expresses [17].

In this study, we aimed to dissect the direct contribution TRPV1 to glucose metabolism. We studied the effect genetic inactivation (*Trpv1* knock out (KO) mice) or pharmacological blockade of TRPV1 (in male Zucker obese rats) on oral glucose tolerance and insulin secretion. Zucker obese (*fa*/*fa*) rats become obese at 4–5 weeks of age due to their hyperphagia and hyperinsulinemia, and then develop diabetes by 14 weeks of age [18]. These animals are commonly used to model human obesity and insulin resistance [19]. For pharmacological blockade, we selected BCTC, a small molecule TRPV1 antagonist that crosses the blood-brain barrier [20]. In rodents, BCTC displays good selectivity for TRPV1 (IC_50_ = 1 nM [21]), although at much higher concentrations it was shown to block TRPM8 (with an IC_50_ of 800 nM [22]) and to activate TRPA1 (with an EC_50_ of 10 µM [23]). BCTC was previously shown to inhibit capsaicin-mediated hyperalgesia, inflammatory hyperalgesia, as well as hyperalgesia after nerve injury [24,25]. Moreover, BCTC was shown to have a positive effect on insulin secretion and insulin sensitivity in *ob*/*ob* mice [26]. Importantly, this BCTC result was later confirmed by using a more selective TRPV1 antagonist by AstraZeneca, AZV1 [27].

## 2. Materials and Methods

### 2.1. Laboratory Animals

Male TRPV1 receptor knock out (*Trpv1^-/-^*; strain B6.129S4-*Trpv1^tm1Jul^*, Stock Number: 003770) and wild-type mice were purchased from Jackson Laboratories (Bar Harbor, ME, USA) and were transported to Denmark at the age of 35 days (5 weeks). At the age of 77 days (11 weeks), the standard chow was changed from normal control diet (D12310, Research Diet Inc., New Brunswick, NJ, USA) to diabetogenic high-fat diet (HFD; D12309, Research Diet Inc.,) on which the mice were kept until the age of 29 weeks. On a caloric base, the HDF consisted of 16.4% protein, 25.6% carbohydrates, and 58% fat, corresponding to an energy content of 23.6 kJ/g. The body weight of the mice was recorded regularly until the age of 170 days (approx. 24 weeks).

Male Zucker obese rats were purchased from Charles River Laboratories (Wilmington, MA, USA) and transported to Novo-Nordisk at the age of 6 weeks.

The rodents were kept under ambient controlled conditions on a normal day light cycle (lights on at 6 am, lights off at 6 pm). The mice were housed as one experimental group per cage, whereas rats were kept in pairs in standard cages (Euro standard Type IV, Techniplast, Scanbur, Denmark), and were given free access to acidified (0.4% citric acid) tap water and standard chow (Altromin, Brogaarden APS, Lynge, Denmark). The animals were trained prior to experiments to minimize stress. Blood samples were obtained from the tail tip capillary from conscious, mildly restrained mice and rats. The rodents were euthanized at the end of the experiments.

Principles of laboratory animal care were followed throughout the study according to EU directive No. 86⁄609. The study design was approved by the Animal Experiments Inspectorate, Ministry of Justice, Denmark.

### 2.2. Oral Glucose Tolerance Test in Trpv1^−/−^ Mice

Before and 16 weeks after placing the mice on HFD, oral glucose tolerance test (OGTT) was performed in the *Trpv1*^−/−^ mice and their wild-type controls. The mice were fasted for 18 h prior to the OGTT. Fasting blood glucose was determined, as described below. Then, glucose at a concentration of 500 mg/mL (Sygehus Apotekerne, København, Denmark) was given orally by gavage (2 g/kg). Samples for measurement of blood glucose were obtained at 30, 60 and 120 min after glucose administration. The area under the blood glucose curves (AUC) of the last OGTT was determined by the trapezoidal method.

### 2.3. Glucose-Stimulated Early Insulin Secretion in Trpv1^−/−^ Mice

One week after the OGTT (17 weeks on HFD), glucose was injected intraperitoneally (i.p.) to stimulate insulin secretion in the *Trpv1*^−/−^ mice and their controls. The mice were fasted for 18 h and then a single i.p. glucose bolus was given (2 g/kg). Samples for the measurement of plasma insulin were obtained 2 min after i.p. glucose.

### 2.4. Determination Of Pancreatic β-Cell Mass and TRPV1-Like Immunoreactivity in Trpv1^−/−^ Mice and their Controls

At the end of the study (after 18 weeks on HFD), the mice were sacrificed; the weight and macroscopic appearance of the abdominal organs (pancreas, liver, epididymal and retroperitoneal white adipose tissue) were recorded; and samples of selected tissues (ventricle of the heart, ileum, liver, abdominal musculature and brown adipose tissue from the neck) were saved for histology. The tissue samples were fixed in 4% buffered paraformaldehyde for 24 h and then embedded in paraffin. Standard (3 µm) tissue sections were stained with hematoxylin and eosin (H&E).

To determine the β-cell mass, sections of the pancreas were deparaffinised and rehydrated; the endogenous peroxidase was blocked by H_2_O_2_ in ethanol; and then the sections were treated with avidin and biotin. Antigen retrieval was performed in a microwave oven in citrate buffer (pH 6) for 3 × 5 min at 90 °C. Sections were stained in an Autostainer (DAKO A/S, Glostrup, Denmark). Non-β cells were labelled by a combination of mouse monoclonal anti-glucagon, rabbit anti-somatostatin, and rabbit anti-pancreatic polypeptide primary antibodies, followed by the combination of biotinylated swine anti-rabbit immunoglobulin G (IgG) and goat anti-mouse IgG and streptavidin peroxidase. The colour was developed with diaminobenzidine (DAB) and NiSO_4_. Pancreatic β-cells were labelled by guinea pig anti-insulin primary and peroxidase-coupled rabbit anti-guinea pig secondary antibodies. The colour was then development with Nova Red (Vector, Burlingham, MA). Biotinylated secondary antibodies were from Jackson Laboratories. All other reagents, including normal sera for blocking, were from DAKO (København, Denmark). Stereological estimations of the mass of β and non-β cells were carried out on two sections cut 200 µm apart (CastGrid V2.0, Olympus, København, Denmark) to control the stage and the data collection. Pancreatic β and non-β cell volumes were estimated by point counting using a grid system with 1 × 256 points. The total mass of β and non-β cells (in mg) was calculated by multiplication by the pancreas weight and the relative pancreas mass (in mg/g body weight) calculated by dividing with the body weight.

To determine TRPV1-like immunoreactivity, following de-paraffination and re-hydration sections of the pancreas were washed in 0.01% phosphate buffer saline and then incubated in 10% normal donkey serum (Jackson Immunoresearch Laboratories, West Grove, PA, USA). Sections were then exposed for 24 h to a primary anti-TRPV1 antibody raised in guinea pigs (Neuromics, Edina, MN, USA) and diluted 1:500. Following washes in PBS, sections were incubated in donkey anti-guinea pig IgG conjugated with tetramethylrhodamine (TRITC) for two hours (Jackson Immunoresearch Laboratories; 1:1000). All the incubations and washes were done at room temperature. As a control for the secondary antibody, the primary antibody was omitted from the buffer. No immunostaining was observed in these controls (not shown). The immunoreaction was observed by a Zeiss epifluorescence microscope using a filter setting for TRITC.

### 2.5. Oral Glucose Tolerance Test and Glucose-Stimulated Insulin Secretion in Zucker Obese Rats Treated with the TRPV1 Antagonist, BCTC

The Zucker obese rats were allocated to study groups based on their body weight the day prior to the administration of the first dose BCTC or vehicle. BCTC was dissolved in vehicle (20% Tween 80 in deionized water) the day before the start of the acute experiment. In the acute experiment, BCTC was given orally at a dose of 15 mg/kg. During the one-week duration of the chronic experiment, BCTC was administered twice-a-day at a dose of 7.5 or 15.0 mg/kg (the dosing volume was 5 mL/kg).

OGTT was carried out immediately after the acute dose of BCTC was given to 6-month-old rats. The test was then repeated three months later with an 8-day (twice-a-day) treatment with BCTC. Prior to the OGTT, the rats were fasted overnight (18 h). At time −30 min (i.e., 30 min before the start of the experiment), samples for blood glucose and plasma insulin were obtained. At time 0 min, BCTC or vehicle was given orally by gavage (1 mL/kg). Glucose (2 g/kg; 500 mg/mL) was given *per os* immediately before BCTC or vehicle administration and blood glucose and plasma insulin levels were determined at time 30, 60, and 120 min after glucose.

### 2.6. Measurement of Mesenteric Adipose Tissue Inflammatory Markers in Zucker Obese Rats Following a Single Dose BCTC

After the eight days dosing study, rats were tested for the presence of inflammatory markers in their mesenteric fat depot. Following an 18 h fasting, rats were given BCTC at 15 mg/kg or vehicle orally by gavage. One hour after treatment, the rats were anaesthetized in isoflurane and decapitated. The mesenteric adipose tissue was quickly removed; transferred to 10X volume pre-chilled RNAlater (Sigma-Aldrich, St. Louis, MO, USA); and stored at −20 °C. The tissues were homogenized in TRIzol at 1 mL/100 mg tissue (InVitrogen, Carlsbad, CA, USA). Total RNA was extracted from 200 mL homogenate as described elsewhere [28].

First-strand cDNA was synthesized using SuperScript III reverse transcriptase and random hexamer primers as described in the manufacturer’s protocol (InVitrogen). cDNA of unknown samples was diluted 1:12 in nuclease-free water (Qiagen, Hilden, Germany). Samples from each cDNA pool were mixed and diluted at 1:6, 1:12, 1:24, 1:48, 1:96, 1:192, and 1:384 in order to create a standard curve for calculation of polymerase chain reaction (PCR) efficiency; only PCR efficiency of 100% (± 1%) and R^2^ between 0.99 and 1.0 were accepted. PCR amplification mixtures (25 µL) contained 12.5 µL of 2X Platinum Quantitative PCR SuperMix-UDG (InVitrogen), 0.625 µL of reverse primer (20 µM), 0.625 µL forward primers (20 µM), 0.625 µL Probe (10 µM) (Universal ProbeLibrary Exiqon A/S, Denmark), and 5 µL diluted cDNA template [29]. Real-time quantitative PCR (RT-PCR) was carried out using the MX3000P PCR machine (Stratagene, La Jolla, CA, USA) with the following cycling parameters: Polymerase activation for 10 min at 95 °C and amplification for 40 cycles of 30 s at 95 °C and 60 s at 60 °C. After amplification, amplicons were validated by gel electrophoresis (E-Gel, InVitrogen).

Relative gene expression of inducible Nitric Oxide Synthase (iNOS) and F4/80 was determined by quantitative RT-PCR (Applied Biosystems, Foster City, CA) using the ABI PRISM 7700 sequence detection system with a vehicle as the calibrator. To normalize expression data, 36B4 were used as internal standard. For each gene, intron-spanning primers were designed using the public domain primer design software from Universal ProbeLibrary Exiqon [29]. Primer and probe numbers were as follows: 36B4 accession no. X15096.1 sense (5′gtgtttgacaatggcagcat 3′), antisense (5′acagacgctggccacatt 3′) and probe Rat#16. iNOS acc. no. NM012611.2 sense (5′ accatggagcatcccaagta 3′), antisense (5′cagcgcataccacttcagc 3′) and probe Rat#71, F4/80 acc. no. XM236797.2, sense (5′ggacttctccaagcctatcgt 3′), antisense (5′cctctcagacttctgctttgg 3′), and probe Rat#26.

### 2.7. Analysis of Blood Glucose and Plasma Insulin

Samples for the measurement of blood glucose were collected in heparinized 5 µL capillary tubes and immediately suspended in 250 µL EBIO buffer (EBIO, Eppendorf, Germany). Glucose was determined using the glucose oxidase method (EBIO, Eppendorf, Germany). Blood for the analysis of plasma insulin was obtained by sampling 70 µL of blood into heparinized 100 µL capillary tubes. These tubes were centrifuged at 4000 rpm for 10 min (4 °C). Plasma (15 µL) was collected and stored at −20 °C until analysis was done, as previously described [25].

### 2.8. Statistical Methods

The two-tailed students *t*-tests was used for the comparison of the group means of all data except for the mean 2-min plasma insulin levels when the two groups were compared by the nonparametric Mann-Whitney U test (due to a statistically significant difference between the variances of the groups). *p* < 0.05 was considered statistically significant.

## 3. Results

### 3.1. Trpv1^−/−^ Mice on High-Fat Diet

The weight and macroscopic appearance of the abdominal organs (pancreas, liver, and white adipose tissue) were similar in the TRPV1 KO and wild-type mice (not shown). Moreover, histologic evaluation of selected tissues (ventricle, ileum, liver, abdominal musculature, and brown adipose tissue from the neck) was normal in both groups (not shown). The β-cell mass was identical in the *Trpv1*^−/−^ and control mice (not shown). As expected, *Trpv1*^−/−^ mice lacked TRPV1-like immunoreactivity in their pancreas whereas the wild-type mice showed TRPV1+ nerve fibers mainly in the connective tissue septa and among the acini. TRPV1-expressing fibres were also seen around or entering the islets (not shown).

**Figure 1:**
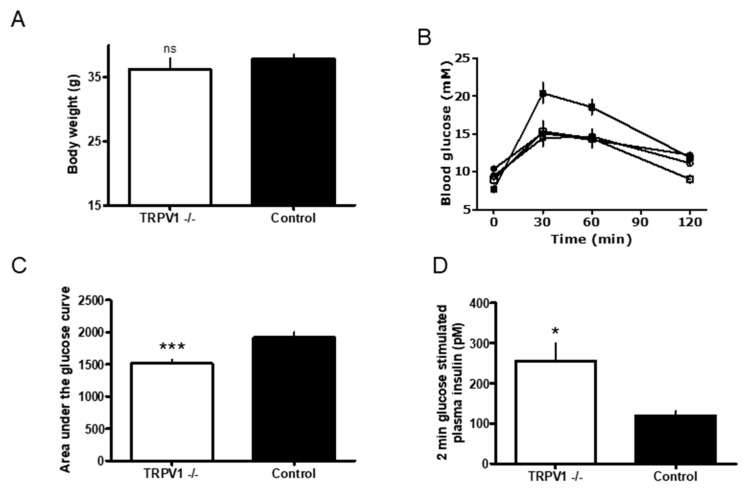
(**A**) The terminal bodyweight of *Trpv1* knock out (KO) (open bar) and wild-type (closed bar) mice on high-fat diet (HFD, open bar). Comparison between the two HFD groups were performed using a two tailed *t*-test, p < 0.05 considered statistically significant. Data are represented as *n* = 10, mean ± Standard Error of the Mean, SEM. (**B**) Blood glucose levels measured in the oral glucose tolerance test (OGTT) in *Trpv1* KO (*n* = 10) and wild-type (*n* = 10) mice. *Trpv1* KO mice were kept on low fat diet (LFD) until the age of 8 weeks (open circles); and then for 16 weeks on high fat diet, HFD (open squares). Wild-type mice on LFD and HFD are represented by open and closed squares, respectively. Glucose was given orally by gavage at time 0 and plasma glucose was assessed prior to this and 30, 60 and 120 min after oral glucose. Blood samples were taken from the tail tips of conscious mice. (**C**) The area under the curve (AUC) analysis of blood glucose levels measured in *Trpv1* KO (open bar) and wild-type (closed bar) mice on HFD during OGTT. Data are from (**B**). *** *p* = 0.0007. (**D**) The glucose stimulated insulin secretion in *Trpv1* KO mice (open bar) and control mice on HFD (closed bar). Mice were given a single intraperitoneal glucose injection and plasma insulin was measured 2 min after the administration of glucose. Samples were obtained, as described above. * *p* = 0.0062.

First, we investigated whether genetic TRPV1 inactivation could reverse weight gain in mice on HFD. The body weight of *Trpv1* KO (*n* = 10) and wild-type (*n* = 10) animals was similar at both the beginning and the end of the study (Figure 1A). The body weight gain curves were mostly parallel between the two groups, with the curve in *Trpv1*^−/−^ mice lying below that of the control animals (not shown). The calculated area under the curve (AUC) in *Trpv1*^−/−^ mice was significantly less than in controls (3,673 ± 97 vs. 3,914 ± 41, *p* = 0.053). The body weight gain was calculated by subtracting the baseline from the actual body weight (δ body weight). The growth curve of the *Trpv1*^−/−^ mice was lower than that of the control mice, and that the AUC of the δ body weight curves was significantly lower in the *Trpv1*^−/−^ mice, as compared to controls (1,190 ± 63 vs. 1,492 ± 64, *p* = 0.004). Taken together, these results suggest that genetic TRPV1 inactivation prevents excessive weight gain associated with ingestion of HFD.

**Figure 2:**
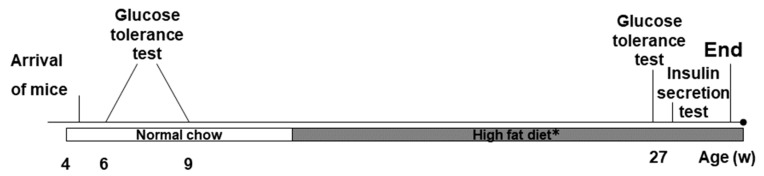
Timeline of the study protocol. *Trpv1* KO and wild-type mice were purchased at the age of weaning and were kept on a standard chow from then until the age of 11 weeks. Oral glucose tolerance test (OGTT) was performed twice in this period at the age of 6 and 8 weeks. From the age of 11 weeks, the mice were transferred to HFD for 16 weeks. A third OGTT was performed at 28 weeks of age by an intra peritoneal glucose-stimulated insulin secretion test. Terminally, at 29 weeks of age, the pancreas was saved for histology.

In the next experiment, we asked whether TRPV1 can play a role in the development of defective glucose homeostasis associated with HFD. We carried out the OGTT test both before and after feeding HFD to *Trpv1*^−/−^ (*n* = 10) and control mice (*n* = 10) for 16 weeks (Figure 2). Before the HFD, the *Trpv1*^−/−^ and control mice (open and closed circles) had similar glucose profiles (Figure 1B). After HDF, however, the control mice (closed squares) had higher levels of blood glucose during the OGTT than the *Trpv1*^−/−^ mice (open squares; Figure 1B). According to the AUC analysis of the data in Figure 1A, the group means of blood glucose levels were comparable between the *Trpv1*^−/−^ and control mice (1,567 ± 53 vs. 1,613 ± 34, *p* = 0.494; not shown). After HFD, however, blood glucose levels were significantly higher in the control animals than in the *Trpv1*^−/−^ mice (1,511 ± 58 vs. 1,915 ± 79; *p* = 0.0007; Figure 1C).

Furthermore, at the end of the OGTT challenge, the *Trpv1*^−/−^ mouse showed no statistical difference (*p* = 0.485) in blood glucose levels before (Figure 1B, open circles) and after HFD (open squares). By contrast, the control mouse on HFD had significantly higher (*p* = 0.002) blood glucose (Figure 1B). Thus, HFD resulted in decreased oral glucose tolerance in the control mice, whereas the *Trpv1*^−/−^ mice retained normal glucose tolerance.

Figure 1D shows the glucose-stimulated plasma insulin secretion in *Trpv1*^−/−^ mice (*n* = 10) and controls (*n* = 10) 2 min after glucose challenge (2g/kg, i.p). The *Trpv1* KO mice (open bar) displayed significantly higher plasma insulin level 2 min after the glucose load (256 ± 43 vs. 119 ± 12 pM; *p* = 0.0062) compared to the control mice (filled bar), suggesting that *Trpv1*^−/−^ mice kept on HFD maintain normal glucose-induced insulin secretion.

### 3.2. The Effect of Pharmacological TRPV1 Antagonism by BCTC on Glucose Metabolism and Mesenteric Adipose Tissue Inflammatory Marker Generation

**Figure 3:**
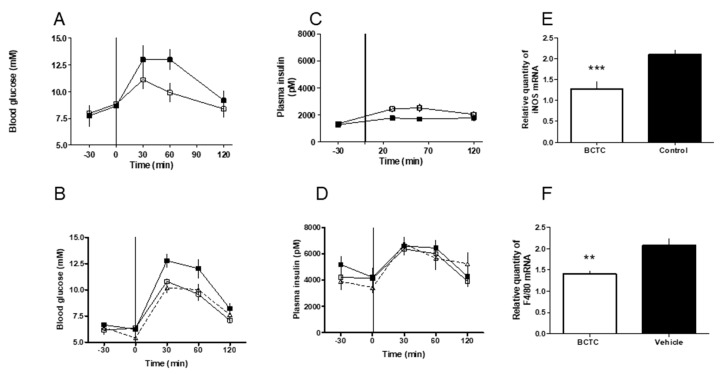
(**A**) The oral glucose tolerance test in male Zucker obese rats after acute oral dosing of the TRPV1 antagonist, BCTC. The rats were given an oral dose of BCTC (15 mg/kg, open squares) or vehicle control (closed squares) by gavage at time −30 min. At time 0, glucose was given orally by gavage and plasma glucose was determined. (**B**) The oral glucose tolerance in male glucose intolerant Zucker obese rats after chronic oral dosing of the TRPV1 antagonist, BCTC. Groups of 9-month-old rats (10 animals each) were given an oral dose of BCTC, 7.5 mg/kg (open squares) or 15 mg/kg (open triangles), by gavage twice-a-day for 8 days. Control rats received vehicle (closed squares). After the last dose (at time −30 min), OGTT was performed. At time 0, glucose was given orally by gavage and plasma glucose was measured in blood samples taken from the tail tips of conscious rats. (**C**) Plasma insulin levels in male Zucker obese rats following acute BCTC (open squares) or vehicle (closed squares) treatment. Insulin was determined in blood samples corresponding to (**A**). (**D**) Plasma insulin levels in male Zucker obese rats following chronic BCTC (7.5 mg/kg, open squares; 15 mg/kg, open triangles) or vehicle (closed squares) administration. Measurements in blood samples taken in (**B**). (**E**) At the age of 9 months, the mesenterial fat depots were removed from BCTC-treated and control rats during anesthesia 30 min after the last dosing. Inducible nitric oxide synthase (iNOS) (**E**) and macrophage marker F4/80 levels (**F**) were determined as detailed in the Methods Section.

In Zucker obese rats, an acute oral dose of BCTC (15 mg/kg, open squares, *n* = 10) improved glucose tolerance compared to vehicle controls (closed squares; *n* = 10, Figure 3A). Similarly, chronic BCTC administration, 7.5 mg/kg (open squares), or 15 mg/kg (open triangles) twice a day for eight days also significantly reduced the increase in blood glucose compared to controls (closed squares; Figure 3B; *p* = 0.001). Insulin secretion was also significantly improved after acute BCTC administration (Figure 3C; *p* = 0.005). Basal insulin levels in 9-month-old rats used in the chronic experiment were, however, markedly elevated and BCTC was not able to further increase the glucose-stimulated insulin secretion (Figure 3D).

In the mesenteric adipose tissue of 9 month-old Zucker rats, 8-day (twice-a-day) treatment with BCTC induced significant reduction in the gene expression of iNOS (from 2.10 ± 0.12 to 1.28 ± 0.17; *p* = 0.001; Figure 3E) and F4/80 (from 2.08 ± 0.16 to 1.40 ± 0.08 vs.; *p* = 0.001; Figure 3F). This suggests that the improvement of glucose metabolism was associated with a reduction of neurogenic inflammation.

## 4. Discussion

The main finding of this study is that pharmacological blockade of TRPV1 is capable of preventing, or even reversing, glucose intolerance in rodent models of T2DM. If this observation holds true in T2DM patients, TRPV1 antagonists may be promising drug candidates to treat impaired glucose tolerance, insulin secretion, and insulin resistance.

Ideally, a novel anti-diabetes drug should normalize blood glucose, reduce bodyweight/obesity, and mitigate the risk of developing cardiovascular disease (CVD) (reviewed in [1]). In the current study, we investigated the effect of TRPV1 blockade on hyperglycemia indirectly by assessing oral glucose tolerance (i.e., the ability to clear orally administered glucose), both as a direct measurement of blood glucose and the insulin response to oral glucose. We also determined the effect of genetic TRPV1 inactivation on weight gain in response to HFD. However, we did not assess the effect of TRPV1 inactivation on CVD risk. Of note, the selective TRPV1 antagonist, AZV1, was reported to improve lipid metabolism in obese (*ob*/*ob*) mice [27]. This observation implies a protective role for TRPV1 antagonism against diabetes-associated CVD.

Hyperglycemia may develop either as a consequence of impaired insulin secretion or decreased insulin sensitivity (insulin resistance). Following glucose challenge, the body is unable to clear the excess glucose; this effect is referred to as glucose intolerance. Elevated fasting blood glucose occurs when excess glucose is not cleared during the night fasting. The progress into frank diabetes is gradual: first, moderately impaired glucose tolerance develops and then fasting hyperglycemia, heralding the onset of frank diabetes. The OGTT is the standard test used in the diagnosis of diabetes-associated glucose intolerance in man. This assay is also widely used in animal models of T2DM to measure the state of glucose intolerance.

In this study, we used OGTT to determine the effect of TRPV1 inactivation on hyperglycemia in *Trpv1* KO and wild-type mice kept on HFD, a model of glucose intolerance. Moreover, we studied the effect of BCTC, a small molecule TRPV1 antagonist [24,25], on oral glucose tolerance in Zucker obese rats, a model of human obesity and insulin resistance [18].

Previously, we determined the role of capsaicin-sensitive, TRPV1-expressing nerves in experimental diabetes (reviewed in [11]). We showed that desensitization of these nerves by capsaicin [14] its ultrapotent analog, resiniferatoxin [30], improves glucose metabolism in experimental animals. When activated, capsaicin-sensitive nerves release proinflammatory and vasoactive neuropeptides—most notably, calcitonin gene-related peptide (CGRP)—that are capable of inhibiting insulin secretion (reviewed in [11]). In theory, reduced levels of these neuropeptides should lead to improved insulin secretion and reduced insulin resistance. Indeed, this was what we found after capsaicin and resiniferatoxin treatment.

TRPV1 is a downstream co-incidence signal detector and integrator on capsaicin-sensitive neurons (reviewed in [31,32]). Various stimuli can act in concert to activate TRPV1 either directly or indirectly, of which heat, protons, and pungent substances (e.g., capsaicin) are the most studied [17,31,32]. The existence of endogenous TRPV1 activators (so-called “endovanilloids”) remains speculative [33]. For example, anandamide (a cannabinoid CB1 receptor ligand) can activate TRPV1 in vitro [34], but it is unclear whether anandamide can reach sufficiently high concentrations in vivo to produce the same effect [33].

Capsaicin desensitization clearly establishes a role of TRPV1-expressing nerves in a biological function. However, it does not necessarily imply that TRPV1 is directly involved since capsaicin renders the whole neuron non-functioning [17]. A well-known example is the rat airway where capsaicin desensitization completely abolishes the neurogenic inflammatory response to cigarette smoke [35]. Irritant compounds in cigarette smoke, however, do not target TRPV1, but rather another receptor, TRPA1, which is co-expressed with TRPV1 on capsaicin-sensitive nerves [36].

In the present study, we wanted to dissect the direct role of TRPV1 in glucose metabolism. First, we induced impaired glucose tolerance in mice by HFD and asked the question whether this could be prevented by the genetic inactivation of TRPV1 (*Trpv1*^−/−^ mice). The HFD-induced impaired glucose tolerance is a useful preclinical model to determine the efficacy of novel anti-diabetic drugs. For instance, allosteric glucokinase activators improve glucose tolerance in this model [37]. In agreement with previous experiments, we found that HFD induced glucose intolerance in the control mice. By contrast, HFD did not impair glucose metabolism in the *Trpv1*^−/−^ animals, suggesting lack of TRPV1 activity prevents the development of obesity-induced disturbance in glucose metabolism.

The original interest in TRPV1 was generated by its postulated role in pain perception [17,31,32]. The effect of genetic TRPV1 inactivation was extensively studied on heat pain perception (reviewed in [31,32]), nociceptive and inflammatory pain [38], and body temperature regulation [39,40,41]. The molecular cloning of the *Trpv1* gene in 1997 had launched major drug discovery programs aimed at identifying potent and selective TRPV1 antagonists [32]. Indeed, a number of TRPV1 antagonists have already entered phase 1 and 2 clinical trials for the indications of migraine, osteoarthritic pain, chronic cough, and atopic dermatitis, just to cite a few examples [32,42]. Some TRPV1 antagonists caused a febrile reaction and/or a modest increase in noxious heat pain threshold, whereas others did not (reviewed in [42]). Other significant adverse effects have not been reported and TRPV1 antagonists were deemed generally safe [43]. That said, recent experiments performed with *Trpv1* KO animals have raised concerns about a possible pro-inflammatory outcome of TRPV1 deficiency [44,45,46,47]. Until the apparent disconnect between animal experiments and human studies are dissolved, patients taking TRPV1 antagonists should be carefully monitored for sepsis, hypertension, renal, and cardiovascular inflammation.

Capsaicin-desensitized rats are smaller and leaner than the vehicle controls [48]. The effect of genetic TRPV1 inactivation on body weight, however, remains controversial with conflicting results. First, *Trpv1*^−/−^ mice were reported to be protected from obesity when kept on HFD [49]. Subsequent studies either detected no effect [50] or described an age-related change: *Trpv1* KO mice were hyperactive and lean when young and then, conversely, “lazy” and fat when they grow old [51]. Moreover, obesity in the old *Trpv1* KO mice was associated with insulin resistance [52]. This observation clearly contrasts to the metabolic health and longevity of TRPV1 deficient mice in a different study [53].

Motter and Ahern [49] reported smaller fat depots and body weights in their *Trpv1* KO animals compared to wild-type controls. Our study agrees with the reduced body weight. We also noticed that *Trpv1*^−/−^ mice gained less weight than their controls when fed HFD. However, we did not notice any difference between *Trpv1* KO and wild-type mice with regard to organ weights or fat depots. This discrepancy may reflect the different fat content in the HDF used by Motter and Ahern (11%) and in our study (58%). Importantly, data from both studies showed a reduction in body weight gain when the TRPV1 is not present, supporting the hypothesis that sensory nerves play a relevant role in the regulation of metabolism, including body weight.

Here, we show that pharmacological blockade by a small molecule antagonist of TRPV1 (BCTC) can recapitulate the phenotype of the *Trpv1* KO mouse fed by HFD in terms of improved handling of glucose. This implies a therapeutic potential for *per os* TRPV1 antagonists in the management of diabetic patients. That said, our results are preliminary with a number of limitations. First, our positive results obtained in animal models of diabetes must be confirmed in diabetic patients. Second, it is unclear if our observations also apply to non-obese diabetics. Thirdly, the long-terms effects of TRPV1 antagonist administration need to be investigated both for beneficial actions (for example, reduced CVD risk, including obesity-induced hypertension [50]) and adverse effects such as impaired noxious heat perception [31,32]).

Another caveat is that we did not measure energy expenditure in our experimental animals. There are reports that dietary capsaicin may keep humans lean [54,55], but no clinical study has been done with regard to the use of TRPV1 antagonists in the treatment or prevention of obesity.

A well-documented effect of TRPV1 blockade is the loss of the neurogenic inflammatory response (reviewed in [17,56]). Of note, obesity-induced pathologies, such as diabetes, seem to be associated with a low-grade inflammatory state [10]. In keeping with this, activation of the inflammatory lipoxygenase pathway has been found in Zucker obese rats [57]. In this study, we assessed the level of two inflammatory markers, F4/80 and iNOS, in the mesenteric fat: Both were significantly reduced after the TRPV1 antagonist BCTC was administered. Our findings suggest that a low-grade inflammatory component may be reduced concomitantly with the improved glucose tolerance. In accordance with this model, TRPV1 antagonists (including BCTC used in our experiments) were shown to block the release of interleukin 1β (IL-1β) and IL-6 from murine macrophages stimulated by lipopolysaccharide, LPS [58]. Likewise, in skeletal muscle, both pharmacological blockade of TRPV1 by antagonists and *Trpv1* knockdown by siRNA suppressed heat-induced IL-6 release [59]. Furthermore, in the human skin the TRPV1 inhibitory peptide, TIP, attenuated the UV-induced increase in the expression of the pro-inflammatory cytokines IL-6 and IL-8 [60]. Taken together these observations support the conclusion that “raised levels of adipokines in wild-type but not TRPV1 knockout mice is in keeping with TRPV1 involvement in stimulating the proinflammatory network that is central to obesity induced hypertension” [50].

[Parenthetically, the converse effect was also reported: namely, TRPV1 activation resulted in the elevation of the expression of IL-1β, IL6, and Tumor Necrosis factor (TNF)-α [61], and genetic ablation of *Trpv1* prevented neuronal activation by IL-1β [62]. The explanation for these seemingly conflicting findings is unknown.]

Interestingly, TRPV1 activation was also reported to improve glucose homeostasis by enhancing glucagon-like peptide-1 secretion in the gut [63]. The phenomenon that TRPV1 activation and blockade produce similar biological outcome via distinct molecular mechanisms is not unprecedented. Indeed, both low-dose capsaicin creams (that activate TRPV1) and high-dose capsaicin patches (that inactivate TRPV1) are in clinical practice to achieve pain relief [17].

We conclude that TRPV1 antagonists may represent a class of novel anti-diabetic drugs that regulate hyperglycemia and prevent weight gain by ameliorating the low-grade inflammation that is seen in diabetes and in obesity.
